# Experimental Gene Therapy with Serine-Histogranin and Endomorphin 1 for the Treatment of Chronic Neuropathic Pain

**DOI:** 10.3389/fnmol.2017.00406

**Published:** 2017-12-08

**Authors:** Stanislava Jergova, Catherine E. Gordon, Shyam Gajavelli, Jacqueline Sagen

**Affiliations:** The Miami Project, Miller School of Medicine, University of Miami, Miami, FL, United States

**Keywords:** gene therapy, serine histogranin, endomorphin 1, neuropathic pain, spinal cord injury, nerve injury

## Abstract

The insufficient pain relief provided by current pharmacotherapy for chronic neuropathic pain is a serious medical problem. The enhanced glutamate signaling via NMDA receptors appears to be one of the key events in the development of chronic pain. Although effective, clinical use of systemic NMDA antagonists is limited by adverse effects such as hallucinations and motor dysfunction. Opioids are also potent analgesics but their chronic use is accompanied by tolerance and risk of addiction. However, combination of NMDA antagonists and opioids seems to provide a stable pain relieve at subthreshold doses of both substances, eliminating development of side effects. Our previous research showed that combined delivery of NMDA antagonist Serine histrogranin (SHG) and endomorphin1 (EM1) leads to attenuation of acute and chronic pain. The aim of this study was to design and evaluate an analgesic potency of the gene construct encoding SHG and EM1. Constructs with 1SHG copy in combination with EM1, 1SHG/EM1, and 6SHG/EM1 were intraspinally injected to animals with peripheral nerve injury-induced pain (chronic constriction injury, CCI) or spinal cord injury induced pain (clip compression model, SCI) and tactile and cold allodynia were evaluated. AAV2/8 particles were used for gene delivery. The results demonstrated 6SHG/EM1 as the most efficient for alleviation of pain-related behavior. The effect was observed up to 8 weeks in SCI animals, suggesting the lack of tolerance of possible synergistic effect between SHG and EM1. Intrathecal injection of SHG antibody or naloxone attenuated the analgesic effect in treated animals. Biochemical and histochemical evaluation confirmed the presence of both peptides in the spinal tissue. The results of this study showed that the injection of AAV vectors encoding combined SHG/EM constructs can provide long term attenuation of pain without overt adverse side effects. This approach may provide better treatment options for patients suffering from chronic pain.

## Introduction

Chronic pain is a major complaint in patients with peripheral or central nerve injury (Cruz-Almeida et al., 2005, 2009; Galluzzi, 2005; Taylor et al., 2010). Clinical and experimental studies have suggested several mechanisms contributing to the development of chronic pain and prospective targets for pharmacological intervention. Although some of these therapeutic strategies provide pain relief, the effects of exogenous pharmacologic agents are usually temporary. In addition, long-term treatments for chronic pain may be limited in sustained effectiveness, dose escalation, development of tolerance and systemic side effects of medication during prolonged use. Contemporary treatment includes pharmacologic and psychological options, but are most appropriate in providing short-term relief (Finnerup and Baastrup, 2012; Yaksh et al., 2017). Therefore, there is a need for improved therapeutic approaches for long-term management of chronic pain.

In order to minimize adverse effects of systemic therapy, it is preferable to target selected mechanisms and pathways involved in the development of chronic pain. Gene therapy can provide suitable tools to achieve this goal. Analgesic peptides may be delivered to specific areas of the nervous system via selective cDNAs, allowing the host cells to serve as local minipumps secreting desired compounds. Direct and sustained delivery of active analgesic agents to specific areas of the CNS circumvents the need for repeat drug bolusing or continual exogenous drug delivery, which may be better suited for long-term pain management.

AAVs are widely considered the top clinically acceptable candidates for gene therapy due to their low toxicity and immunogenicity, as well as ability to provide continued transgene expression and long-term production of desired therapeutic molecules (Kotterman and Schaffer, 2015; Albert et al., 2017; Naso et al., 2017; Wells, 2017). Although this approach is still in nascent stages for chronic pain management, the use of AAV-mediated delivery of potential antinociceptive molecules have shown promising outcomes in rodent peripheral neuropathic pain models (Eaton et al., 2002; Beutler et al., 2005; Beutler and Reinhardt, 2009; Pleticha et al., 2014; Guedon et al., 2015). Screening of several AAV2 serotypes in our lab showed AAV2/8 as an optimal vector for intraspinal delivery of recombinant cDNA.

Glutamate excitotoxicity via signaling through NMDA receptors is one of the key events in the onset and maintenance of the neuropathic pain after peripheral or central nerve injury. Supporting this hypothesis, studies have shown decreased hyperexcitability and cutaneous hypersensitivity with intrathecal administration of NMDA receptor antagonists (Sotgiu and Biella, 2000; Suzuki et al., 2001). Unfortunately, the clinical use of systemic NMDA antagonists is dose-limited by adverse effects such as hallucinations and motor dysfunction (Hawksworth and Serpell, 1998; Galluzzi, 2005). Previous studies have shown that the naturally occurring peptide histogranin (Lemaire et al., 1993) and its stable analog Serine-Histogranin (SHG) have NMDA receptor antagonist activity and do not interfere with locomotion (Lemaire et al., 1995; Prasad et al., 1995; Hama et al., 1999; Hama and Sagen, 2002; Hentall et al., 2007). Furthermore, data has shown marked attenuation of hyperalgesia and allodynia with the spinal introduction of SHG through intrathecal injection or via cell or gene therapy (Siegan et al., 1997; NasiriNezhad and Sagen, 2005; Gajavelli et al., 2008; NasiriNezhad et al., 2015; Jergova et al., 2016a,b).

Opioids are potent analgesics and as such have been used in treatment of chronic pain; however their chronic use is accompanied by tolerance and risk of addiction (Sallerin-Caute et al., 1998; Przewłocki and Przewłocka, 2001; Ossipov et al., 2004). NMDA antagonists have been shown to prevent development of morphine tolerance and allow prolonged administration of opioids (Trujillo and Akil, 1991, 1994).

Therefore, the combined administration of NMDA antagonist and opioid receptor agonist may be beneficial especially for the long-term pain management. Endomorphins (EM) are endogenous opioid peptides highly selective for μ-opioid receptors (Zadina et al., 1999). Their two isoforms have a distinct anatomical distribution, with endomorphin1 (EM1) present mainly in the brain and EM2 in the spinal cord. They also differ in the analgesic properties with EM1 more potent in attenuation of inflammatory and neuropathic pain (Przewłocka et al., 1999; Przewłocki et al., 1999; Przewłocki and Przewłocka, 2001; Hama and Sagen, 2014), while lower analgesic efficacy in the model of inflammatory pain and no effect in neuropathic pain are reported for EM2 (Hama and Sagen, 2014). Our previous experiments showed potentiation of the analgesic effect of intrathecally delivered EM1 by SHG in the formalin model (Hama and Sagen, 2014). Our recent study with gene constructs encoding either peptide showed that coinjection of EM1 and SHG is beneficial for alleviation of spinal cord injury induced pain (NasiriNezhad et al., 2015). The goal of the present study was therefore to design recombinant cDNA encoding both SHG and EM1 analgesic peptides in several combinations in order to simplify the delivery of both analgesic peptides, to reduce the amount of viral particles needed for injection and to identify the most efficient combination for the alleviation of pain-like behavior in our models.

## Materials and Methods

### Experimental Plan

The flow of experiments reported in this study is as follows: (1) evaluation of analgesic potency of single and multi-copy SHG and single EM1 constructs in a peripheral nerve injury model (CCI) and spinal cord injury model (SCI); (2) design and engineering of a combined construct encoding SHG and EM1; 1SHG/EM1 and 6SHG/EM1; (3) transduction of combined plasmids into HEK cells to confirm infectivity and peptide production; and (4) production of AAV viral particles encoding the combined constructs and evaluation of the analgesic effect of the recombinant rAAV constructs in CCI and SCI models.

### Construction of the Combined SHG-EM1 Plasmids

Lentiviral particles encoding EM1 and SHG have been previously generated in our lab and evaluated in the pain models (NasiriNezhad et al., 2015). In the current study we initially attempted to engineer all constructs into the lentiviral backbone. However, while successful with single SHG and EM1, due to low yield of lentiviral plasmids with other recombinant genes we used AAV plasmid to generate 6SHG constructs and the combination SHG/EM constructs. Our previous screenings of different serotypes of AAV vector for transduction of neuronal cells showed the hybrid AAV2/8 as the most efficient in the spinal dorsal horn. To evaluate possible differences in the transduction efficiency between lenti and AAV plasmids, control plasmids with eGFP reporter gene were initially used for the transduction of the HEK 293T cells (below). All viral particles were generated by Viral Vector Core at Miami Project.

#### EM1 and 1SHG

The generation and characterization of the EM1 and SHG constructs has been previously published (Gajavelli et al., 2008; NasiriNezhad et al., 2015). Briefly, EM1 and SHG protein sequences were reverse translated and restriction sites (BglII/SalI and BglII/ XbaI, respectively) were added on 5’ and 3’ of the cDNA. Oligos were synthesized, purified, annealed and subcloned into bacterial vector, linearized with an appropriate restriction endonuclease and subcloned into pRRL lentiviral vector in frame with mRFP as a reporter gene. ppNGF-β signal sequence was added to achieve production of secretable peptides (Duplan et al., 2004).

#### 6SHG

SHG multimers were generated by adding BglII restriction sites at 5’SHG oligo sequence using PCR (Jergova et al., 2016a). BglII flanked SHG sequences were annealed to generate SHG multimers and subcloned into a bacterial vector with single SHG. Clones were screened for the correct orientation of each SHG copy within the multimers (Genewiz). We were able to isolate a clone with six copies of SHG and to generate AAV2/8 viral particles using this plasmid.

#### 1SHG/EM and 6SHG/EM

Formation of the combined SHG-EM1 construct was completed by replacing the mRFP coding sequence with the EM1 construct. The mRFP cDNA was excised from the SHG vector using restriction sites XbaI and SalI. Excision was confirmed via gel electrophoresis and the SHG construct purified using gel electrophoresis isolation and gel extraction. Oligos of EM1 flanked by XbaI-SalI restriction sites were synthesized, purified and subcloned into AAV2/8 vector in frame with SHG cDNA.

A similar strategy was initially attempted to generate a 6SHG/EM1 construct using 6SHG AAV vector. However, this approach yielded low amounts of recombinant AAV vector (rAAV), insufficient for producing viral particles. Therefore the subcloning steps were done using bacterial vector pBS (Addgene). 6SHG cDNA was excised by BamHI-XbaI and subcloned into pBS vector into open reading frame to get additional restriction sites 5’ClaI 3’KpnI required for subcloning into AAV vector. Oligos of EM1 flanked by AgeI-KpnI restriction sites were synthesized, annealed, PCR amplified and subcloned into pBS in frame with 6SHG. The presence and correct orientation of SHG and EM constructs were confirmed by sequencing (Genewiz). Bacterial colonies with the correct plasmids were further grown to obtain sufficient amount of plasmids to be used for subcloning into AAV vector. Plasmids were isolated and SHG-EM constructs were excised by ClaI-KpnI restriction enzymes, purified and subcloned into AAV vector. Final AAV plasmids were subsequently used for production of AAV viral particles by the Miami Project Viral Vector Core.

### Transgene Viral Expression *in Vitro*

The stability of designed recombinant plasmids and the transduction efficiency of lenti GFP and AAV2/8 GFP plasmids were tested with the HEK293 cell line. Cells were transduced with plasmids using a Lipofectamine kit. SHG and EM1 peptides were detected in the supernatant obtained from cultured cells using the FLISA method. Briefly, HEK293 cell lines were grown in DMEM/F12 medium (Invitrogen, Carlsbad, CA, USA) supplemented with 10% fetal bovine serum (FBS) and antibiotics. At 70% confluency the cells were transduced with GFP control plasmids (pLenti and AAV2/8) or recombinant 1SHG/EM and 6SHG/EM plasmids by 10^8^ transducing units per ml (TU/ml) based on viral coat protein p24 ELISA assay. Five days after transduction, the cells were plated into petri dishes at a concentration 50 × 10^6^ cells in 10 ml of media and incubated for 1 day. Media was collected and used for evaluation of SHG and EM1 presence using the FLISA method. Total concentration of peptides in the sample was assessed by BCA kit (Thomson Scientific). Samples were then plated in 96 well plates with equal concentration of peptides, captured overnight at 4°C, incubated in blocking solution, followed by primary antibodies at different dilutions (SHG, 1:100–1:500, 21st Century Biochemical; EM-1, 1:10–1:50, gift from Dr. James Zadina, Tulane University) and secondary antibodies (Li-cor 800 IRDye), according to the FLISA Li-cor protocol. The signal was scanned by an Odyssey infrared scanner. After confirming transduction capability, 1SHG/EM1 and 6SHG/EM1 vectors were subcloned into AAV vector by the in-house Miami Project Viral Vector Core.

For evaluation of GFP expression, 5 days after transduction, cells were plated into 24 well plates in concentrations of 1 × 10^4^ cells per well, cultured for 1 day, fixed with 4% paraformaldehyde and processed for immunocytochemistry. Plates were washed with 0.1 M PBS, incubated in 5% normal goat serum, followed by GFP primary antibody (1:200, Sigma) for an overnight incubation. After a brief wash cells were incubated in the secondary antibody (1:250, Alexa Fluor 488, Invitrogen), washed and coverslipped.

### Animals

Male Sprague–Dawley rats weighing 120–140 g at the initiation of the studies were used. Rats were allowed 3–5 days to acclimate to the animal facility which was on a 12 h light/dark cycle. Prior to surgery and following surgery, rats were allowed free access to food and water. For surgical anesthesia, rats were rendered unconscious with 4%–5% isoflurane in O_2_ and maintained on 2%–3% anesthesia. Aseptic technique was used throughout each surgical procedure. All animal procedures followed NIH guidelines and were approved by the University of Miami Institutional Animal Care and Use Committee. GraphPad RandomNumbers calculator was used to randomly assign animals into CCI or SCI groups and for the treatments within the groups. Sigma Stat software was used to estimate the sample size for each group. Input data were used based on our experiences with the pain models and procedures. Using the power analysis calculator the calculated sample size was 7–10 for treatment groups and 4–7 for control groups. Only the animals that showed presence of pain-like behavior were included in subsequent experiments. Animals with any post-injury complication, especially in the SCI group, were excluded from the next steps. An attempt was made to keep the same number of animals per group for more reliable statistical comparison. *N* = 8 and *n* = 6 were the target numbers for treatments or control GFP groups respectively, with *n* = 54 per injury group (CCI or SCI).

### Surgeries

All the surgical procedures were performed using aseptic technique in a special room designed for surgery purposes.

#### Chronic Constriction Injury (CCI)

For chronic constriction injury, under anesthesia, the left sciatic nerve was exposed at the level of mid-thigh. Four 4-0 chromic gut ligatures were loosely tied around the sciatic nerve with about 1 mm spacing between them. The muscle wound was sutured shut and the skin was closed with veterinarian-grade cryoacrylate. Rats recovered on heating pads and returned to cages (Bennett and Xie, 1988; Jergova et al., 2012).

#### Spinal Cord Injury (SCI)

For SCI, under anesthesia, a laminectomy was performed at T6–T8. The spinal cord was compressed by a 20 g microvascular clip (Harvard Apparatus, MA, USA) for 60 s After a 1 min compression, the clip was removed carefully (care was applied not to damage the dura or spinal nerves), the muscles sutured with 4–0 chromic gut (Ethicon Inc., Somerville, NJ, USA) and wound clips were used to close the skin. From the day after surgery the bladder was manually expressed in the morning and afternoon until the animals were able to express their bladders independently. Gentamicin (2 mg/kg, i.p.) was injected for 7 days post surgery to prevent bladder infections (Bruce et al., 2002; Hama and Sagen, 2007; Jergova et al., 2016a).

#### Intraspinal and Intrathecal Injections

Animals were randomly allocated into treatment and control groups. For characterization of the analgesic effect of 1SHG, 6SHG and EM1 viral constructs and the compound constructs SHG/EM, 1 week post CCI and 4 weeks post SCI time points were chosen as the injection windows, respectively. Since neuropathic pain-like symptoms are long-lasting in the SCI clip compression model, there is opportunity to assess delayed treatments which may more closely mimic the clinical scenario for SCI neuropathic pain intervention.

##### Intraspinal

For all intraspinal injections, spinal cords at the level of L3/L4 were exposed by laminectomy. Viral constructs were injected either unilaterally (CCI) on the injured side or bilaterally (SCI) in spinal cord in volumes of 1.0 μl at 0.2 μl per min rate in titers of 10^6^ (AAV) and 10^8^ (Lenti). Injections were done stereotaxically (KOPF) at a depth of 0.3 mm from the dorsal border and 0.7 mm from the midline using a glass needle attached to a 10-μL Hamilton syringe mounted on a microinjector. To prevent backflow, the needle was kept in its place for 1 min after the termination of the injection. To maximize reproducibility, injections were performed by the same surgeon. The overlying muscles were sutured by Vicryl (Ethicon) and the skin was closed with wound clips. Animals were allowed to recover at 37°C for 24 h, after which time they were returned to the animal care facility.

##### Intrathecal

Intrathecal injection of SHG antibody (5 mg/ml, custom synthesized by 21st Century Biochemicals) and Naloxone (0.1 mg/ml) were used in some SCI animals (*n* = 6/group) to further analyze the effects of the compound construct on pain-like behavior. An intrathecal catheter (7.5–8 cm; ReCathCo, PA, USA) was threaded through a slit in the atlanto-occipital membrane down the intrathecal space and secured to the neck muscles with sutures under 2%–3% isoflurane/O_2_ anesthesia as described previously (Yaksh and Rudy, 1976; Hama and Sagen, 2009). This procedure brings the tip of catheter to the lumbar spinal segments. Rats were allowed to recover at least 3 days following intrathecal surgery prior to use in experiments. Drugs were dissolved in saline and injected in 5 μl volumes, followed by 5 μl flush with saline.

### Behavioral Testing

Animals were observed daily for the overall wellbeing post-surgery. Weight was recorded weekly. All the behavioral tests were done by evaluators who were unaware of experimental groups. For the assessment of tactile and cold allodynia, the rats were placed on a metal mesh covered with a plastic dome (13 × 17 × 28 cm) for at least 20 min before testing. Animals were tested before any injury (baseline), before intraspinal injection of vectors (post injury) and then weekly up to 6 weeks in the CCI model and 12 weeks in the SCI model. Since spasticity or hyperreflexia in SCI animals may interfere with behavioral results and interpretation, all behavior test endpoints were based on higher CNS, non-reflexive signs of discomfort elicited by applied stimulus, such as turning head toward the stimulus, shaking and licking stimulated paw, vocalizing and moving away from the stimulus. At least one of these signs had to be present in order to record a paw withdrawal upon stimulation as a positive (pain related) response. Previous findings in our lab have indicated that the inclusion of these higher center responses correlates well with more complex cognitive outcome measures for nociception in SCI animals (Dugan and Sagen, 2015a,b).

#### Tactile Allodynia

The thresholds for mechanical allodynia were measured with a series of von Frey filaments using the up-down method (Chaplan et al., 1994). The filaments were applied to the plantar skin of the hindpaw and bent slightly. Withdrawal of the paw, usually followed by licking of the paw or body movement, was recorded as a positive response. Eight specific calibrated von Frey filaments were used via the up-down method to determine the withdrawal threshold. The filaments with 0.25 g and 15 g force were selected as lower and upper limit, respectively. A hind paw withdrawal from the filament led to the use of the lower force filament. A lack of response led to the use of the next higher force filament. If the strongest hair did not elicit a response, the threshold was recorded as 15 g. Both hind paws were tested with about 5 min pause between subsequent tests.

#### Cold Allodynia

Cold allodynia was evaluated by counting the number of foot withdrawal responses after application of an acetone drop to the plantar surface of the paw. Usually the normal rat did not respond to acetone application, but the injured rats showed nociceptive responses, such as foot shaking or biting. The testing was repeated five times with an interval of approximately 3–5 min between each test for both hind paws. The response frequency to acetone was expressed as a percent response frequency ([number of paw withdrawals/number of trials] × 100; Choi et al., 1994).

### Neurochemical and Immunocytochemical Evaluations of Spinal Cord Tissue

To confirm the expression of transgene, spinal cord samples were prepared for neurochemical or immunocytochemical evaluations at the end of behavioral experiments (6w post CCI or 12w post SCI, *n* = 3/group). For neurochemical analyses, spinal cord samples were dissected at lumbar regions, dorsal parts separated and frozen on dry ice. Samples were homogenized in RIPA lysis buffer (Santa Cruz) and protein concentration was estimated by BCA kit (Thomson Scientific). Homogenates were processed for Fluorescence-linked immunosorbent assay (FLISA). Briefly, samples were loaded in 96 well plates at the equal concentration, captured overnight and incubated in the primary antibodies (SHG, 1:100–1:500, 21st Century Biochemical; EM-1, 1:10–1:50, gift of Dr. Zadina) and secondary antibodies (Li-cor 800 IRDye), according to FLISA Li-cor protocols. The signals were scanned by an Odyssey infrared scanner.

For immunocytochemical analyses, animals were deeply anesthetized and intracardially perfused with 0.9% saline followed by 4% paraformaldehyde in 0.1 M phosphate buffer. Spinal cords were removed and post-fixed for 12 h in the same fixative and transferred to 25% sucrose for cryoprotection. Free floating cryostat sections were prepared and processed according standard immunohistochemical protocols. Sections were incubated in 5% normal goat serum for 2 h followed by overnight incubation with primary antibodies NeuN (1:200, Chemicon), SHG (1:100, 21st Century Biochemicals), and EM-1 (1:10, courtesy of prof. Zadina et al., 1999). After washing, sections were incubated in the appropriate secondary antibodies (Alexa Fluor 488 goat anti-mouse and Alexa Fluor 594 goat anti-rabbit). DAPI staining was use to visualize cell nuclei. Sections were then washed, mounted on gelatin-covered slides, air dried and coverslipped. Images were analyzed by confocal microscope (Spectral Confocal Microscope Fluoview 1000).

### Statistical Analysis

Data were analyzed by Sigma Stat software to detect statistical significances. The results are presented as means ± SEMs. Data were compared among groups using two-way analysis of variance with repeated measures (RM ANOVA) or one way ANOVA where applicable, followed by the Bonferroni *post hoc* test. Statistical significance was assumed when *p* < 0.05.

## Results

### Analgesic Effect of Single Gene Constructs

The analgesic effect of the intraspinal injection of pLenti_1SHG, pLenti_EM and AAV2/8_6SHG constructs was evaluated after CCI and SCI-induced chronic pain and compared with the control CCI and SCI animals injected with AAV2/8_GFP. All animals involved in this experiment underwent CCI or SCI. Animals exhibiting the presence of tactile and/or cold allodynia at the beginning of behavioral evaluation (1 week post CCI, 4 weeks post SCI) with paw withdrawal threshold values ≤8 and responses to acetone ≥50% were used in the subsequent experiments (for SCI rats the exclusion criteria applied if none of the hind paws reached the threshold value; *n* = 54). Viral vectors encoding analgesic genes or GFP were injected at week 1 post CCI into the ipsilateral lumbar spinal cord and 4 weeks post SCI bilaterally into lumbar spinal cord.

#### CCI (Figures 1A,B)

In the tactile allodynia test, animals treated with analgesic genes showed progressive increase in paw withdrawal threshold after the injection of the construct compared to control GFP group (*F*_(*df*3,18)_ = 1.449; *P* = 0.0068). Towards the end of the experiment, by week 6 post injury, the effect was slightly reduced. Significant differences compared to GFP group were observed at 2 weeks post injection (3 weeks post injury) in the 1SHG group (^+^*p* < 0.05) and at 2 and 3 weeks post injection in the 6SHG group (^#^*p* < 0.05). Animals treated with EM1 alone showed a similar trend in increasing paw withdrawal threshold, but this did not reach statistical significance compared to control GFP group. The single construct treatment was more potent in attenuating cold allodynia. Significant differences were observed from week 1 post injection up to week 4 (overall *F*_(*df*3,18)_ = 1.016; *p* < 0.0001). The responses to acetone were significantly reduced in all treatment groups with the peak effects observed at 2 weeks post injection (^++^*p* < 0.01 for 1SHG vs. GFP; ^###^*p* < 0.001 for 6SHG vs. GFP; **p < 0.01 for EM vs. GFP).

**Figure 1:**
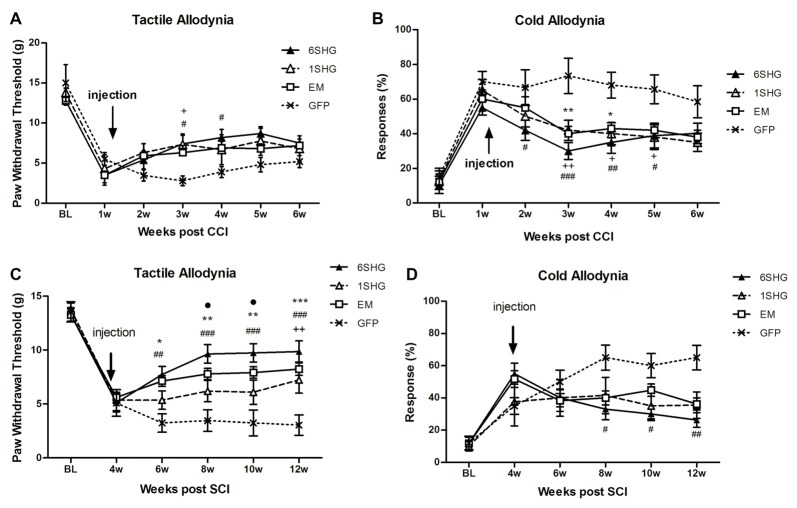
Analgesic effect of individual gene constructs in chronic constriction injury (CCI) **(A,B)** and spinal cord injury (SCI) **(C,D)** animals. **(A)** Injection of 1SHG and 6SHG constructs attenuated tactile allodynia compared to GFP controls at 2 and 3 weeks post injection. **(B)** Cold allodynia was attenuated in all treated animals compared to GFP control at one through 4 weeks post injection. **(C)** Stronger analgesic effect of the gene construct was observed in SCI model with attenuation of tactile allodynia in all treated animals up to 8 weeks post injection. **(D)** Similarly, in cold allodynia test marked drop in cold sensitivity was observed in treated animals (RM ANOVA; ^+^*p* < 0.05, ^++^*p* < 0.01 for 1SHG vs. GFP; ^#^*p* < 0.05, ^##^*p* < 0.01, ^###^*p* < 0.001 for 6SHG vs. GFP; *p < 0.05, **p < 0.01, ***p < 0.001 for EM vs. GFP; ^•^*p* < 0.05 for 6SHG vs. 1SHG).

A partial recovery observed in the model of CCI over time (starting around 5–6 weeks post-CCI surgery) may accounts for the lack of statistical significance of the observed effects in the treated animals towards the latter time points of the experiments.

#### SCI (Figures 1C,D)

SCI induces development of pain-like behavior in animals after their partial recovery from the initial hind paw paralysis. Animals were left to recover from the injury and the behavioral tests started at 4 weeks post injury. The constructs were delivered at the same week to target established chronic pain. Significant attenuation of tactile allodynia was observed in all treatment groups up to 8 weeks post injection (12 weeks post injury) compared to the control group (*F*_(*df*3,15)_ = 2.634; *P* < 0.0001). The most effective attenuation of tactile allodynia was observed in the 6SHG group, with significantly different values from 1SHG group at 8 and 10 week post injury (^•^*p* < 0.05). Attenuation of cold allodynia was observed in all treated animals as well (*F*_(*df*3,15)_ = 1.741; *P* = 0.0016) although the effect was significant only in 6SHG group compared to GFP group (^#^*p* < 0.05; ^##^*p* < 0.01).

### Engineering of Compound SHG/EM Constructs

**Figure 2:**
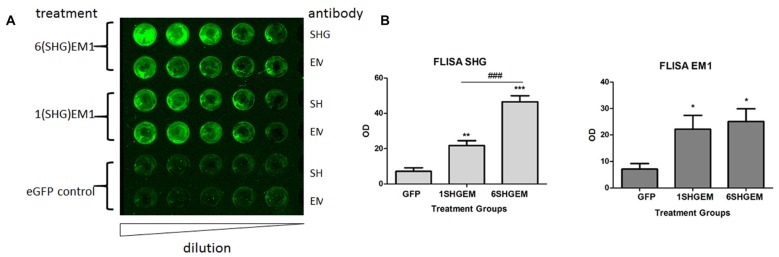
Fluorescence-linked immunosorbent assay (FLISA) analysis of HEK 293 supernatant for the presence of serine histrogranin (SHG) and EM recombinant peptides after transduction with 1SHG/endomorphin1 (EM1), 6SHG/EM1 and GFP gene constructs. **(A)** SHG and EM1 peptides detected in samples from compound gene constructs transduced cell cultures. No signal detected in the control samples from GFP transduced cells. **(B)** The level of SHG in 6SHG/EM samples was significantly higher than in 1SHG/EM samples as expected. The level of EM was comparable between samples (ANOVA; *p < 0.05, **p < 0.01, ***p < 0.001 vs. GFP control; ^###^*p* < 0.001 for 6SHG/EM vs. 1SHG/EM).

To evaluate the potential for enhancement of the analgesic effect of SHG constructs by targeting the opioid signaling pathway, we engineered a compound construct of a single and multiple SHG with EM1. Since the single gene constructs were designed using lentiviral particles, to evaluate possible differences in the transduction efficiency between plasmids, eGFP reporter gene were initially used for the transduction of the HEK 293T cells. Transduction efficiency has been evaluated by immunocytochemistry based on the presence of GFP signal in cells. No significant differences in the number of GFP+ cells have been found. To confirm stability and proper production of SHG and EM1 recombinant peptides, HEK293 cells were transduced with either 1SHG/EM1 or 6SHG/EM1 plasmids. SHG and EM1 peptides were detected in the supernatant obtained from cultured cells using the FLISA method (Figure 2A). Signals for both SHG and EM were significantly higher when using the appropriate antibodies respectively compared to control GFP supernatant (*p < 0.05, **p < 0.01, ***p < 0.001 vs. control). Stronger SHG signal was detected in the samples from 6SHG/EM1 transduced cells compared to 1SHG/EM1 as would be expected (^###^*p* < 0.001). The EM1 signal was comparable between samples (Figure 2B).

### Analgesic Effect of Compound Gene Constructs

After confirming transduction capability, 1SHG/EM1 and 6SHG/EM1 plasmids were used to produce viral rAAV_2/8 particles by the in-house viral vector core. The analgesic potential of constructs was then assessed in CCI and SCI models using the same injection window as for the single constructs. At the end of behavioral evaluations, data from single and compound constructs groups were also retrospectively compared at selected time points as an indication of potential differences in onset or potency.

#### CCI (Figures 3A,B)

Strong antinociceptive effects in both tested modalities were observed when using compound constructs. Tactile allodynia was attenuated in the treated animals at 3 weeks post injection and the effect sustained for the following 2 weeks (*F*_(*df*2,12)_ = 2.001; *P* < 0.0001). Although the analgesic effect was not significantly different between 6SHG/EM1 and 1SHG/EM1 groups, there was a tendency towards consistently higher von Frey thresholds in the 6SHG/EM1 group. Cold allodynia was significantly attenuated in both treatment groups starting at 1 week post injection, with the anti-allodynic effects observed up to 5 weeks post injection (*F*_(*df*2,12)_ = 2.415; *P* < 0.0001). Both treatment groups showed these significant effects compared to the control group (^+^*p* < 0.05, ^++^*p* < 0.01 1SHG/EM1 vs. GFP; ^##^*p* < 0.01, ^###^*p* < 0.001 6SHG/EM1 vs. GFP).

**Figure 3:**
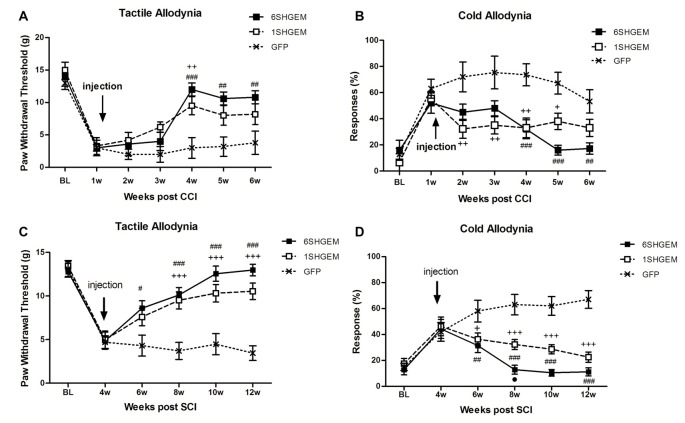
Analgesic effect of compound gene constructs in CCI **(A,B)** and SCI **(C,D)** animals. **(A)** Tactile allodynia was attenuated at 3 weeks post injection in both 1SHG/EM1 and 6SHG/EM1 treatment groups and the effect sustained up to 5 weeks post injection. **(B)** Cold allodynia was attenuated in both treatment groups as well with effect observed 1 week post injection up to 5 weeks. **(C,D)** In SCI groups, both gene constructs demonstrated analgesic efficacy in tactile **(C)** and cold **(D)** allodynia tests with effect observed 2 week post injection and sustained till the end of experiment (RM ANOVA; ^+^*p* < 0.05, ^++^*p* < 0.01, ^+++^*p* < 0.001 1SHG/EM1 vs. GFP; ^#^*p* < 0.05, ^##^*p* < 0.01, ^###^*p* < 0.001 6SHG/EM1 vs. GFP).

#### SCI (Figures 3C,D)

Compound constructs significantly attenuated tactile (*F*_(*df*2,10)_ = 5.2; *P* < 0.0001) and cold allodynia (*F*_(*df*2,10)_ = 6.584; *P* < 0.0001) with the effects observed at 2 week post injection, and this was sustained up to 8 weeks post injection (12 weeks post injury, the end of the experiment). Both groups were significantly different from the control GFP group, with the trend of stronger effects in 6SHGEM group compared to 1SHGEM, especially towards the latter time points of the study, when nearly complete reversal of both tactile and cold allodynia were observed in the 6SHGEM treatment groups (^+^*p* < 0.05, ^+++^*p* < 0.001 1SHG/EM1 vs. GFP; ^#^*p* < 0.05, ^##^*p* < 0.01, ^###^*p* < 0.001 6SHG/EM1 vs. GFP).

### Comparison of Single and Compound Gene Treatment

To compare the effect of the single and compound gene treatment data from both experiments were pooled at the baseline, pre injection time points and at the 5 weeks (CCI) or 6 weeks and 8 weeks (SCI) post injection. The values in GFP groups were averaged as there were no significant differences between GFP groups used as a control for the injection of single or compound constructs. At each time point values were compared between treatment groups using one way ANOVA. These comparisons suggested that the 6SHG/EM1 construct is the most efficient in reducing pain-like behavior induced by CCI or SCI injuries.

#### CCI (Figures 4A,C)

In both tactile and cold allodynia tests no differences were observed at the baseline or pre-injection time points. At 5 weeks post injection the 6SHG/EM1 showed significant anti-allodynic effect compared to the control GFP group (**p < 0.01).

**Figure 4:**
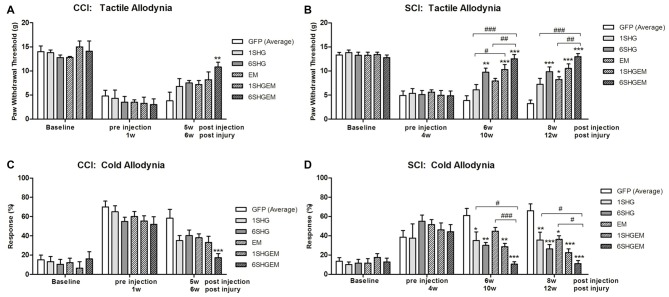
Comparison of the analgesic effect of singe and compound gene constructs at 5 weeks post injection in CCI animals **(A,C)** and 6 and 8 weeks post injection in SCI animals **(B,D)**. In both groups and both pain modalities, no differences were observed at the baseline or pre-injection time points. In CCI group at 5 weeks post injection the effect of 6SHG/EM1 was significantly different from GFP controls (ANOVA; **p < 0.01). In SCI group all treated animals demonstrated reduction in hypersensitivity with significant differences compared to control GFP groups as well as inter-treatment differences. The effect was observed up to 8 weeks post injection of the constructs with 6SHG/EM1 as the most efficient construct reducing pain-like behavior (ANOVA; *p < 0.05, **p < 0.01, ***p < 0.001 vs. GFP, ^#^*p* < 0.05, ^##^*p* < 0.01, ^ ###^*p* < 0.001 between treatments).

#### SCI (Figures 4B,D)

In the tactile allodynia test (B) at the comparable time point to CCI group (6 weeks post injection), differences from GFP were observed in 6SHG, 1SHG/EM1 and 6SHG/EM1 groups (*p < 0.05, **p < 0.01, ***p < 0.001) with significant intra-treatment differences between 1SHG/EM1 or 6SHG/EM1 and 1SHG groups, and 6SHG/EM1 and EM1 groups respectively (^##^*p* < 0.01; ^###^*p* < 0.001). The analgesic effect was still present at 8 weeks post injection with similar group differences. In the cold allodynia test (D) no differences at the baseline or pre-injection points were detected. At both time points (6w and 8w) almost all groups were significantly different from GFP group (*p < 0.05; **p < 0.01; ***p < 0.001) with 6SHG/EM1 different from 1SHG and EM1 groups, respectively (^#^*p* < 0.05; ^###^*p* < 0.001).

Overall, the behavioral tests suggest that compound constructs with the combination of the analgesic genes targeting glutamate and opioid pathways are potent and stable in reducing pain-related behavior compared to the single treatment. No significant side effects were observed in the treated animals. Minor post-injury related issues (bladder inflammation) were addressed as needed. In general, animals were in a good physical condition, without any signs of constipation, diarrhea, lethargy or weight loss as would be expected from the prolonged treatment with high dose of opioids.

### Intrathecal Injection of Anti-SHG and Naloxone

**Figure 5:**
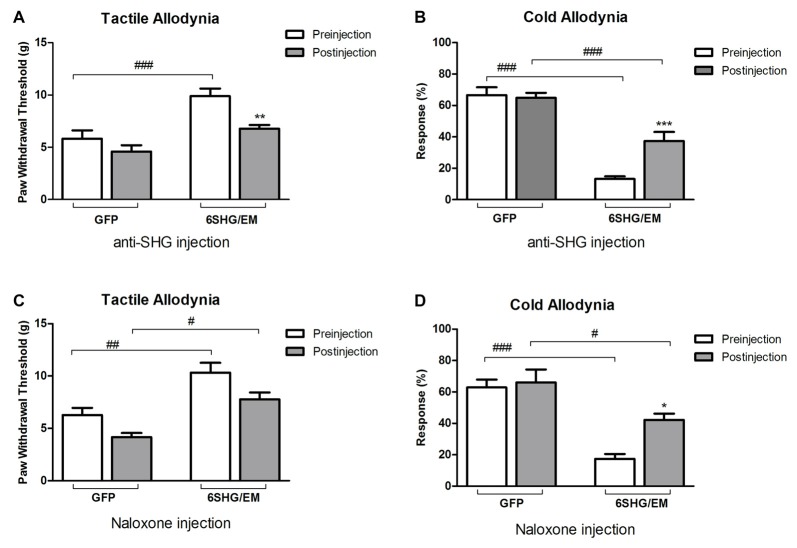
Pharmacological attenuation of the 6SHG/EM1 analgesic effect. Intrathecal injection of anti-SHG (5 mg/ml; **A,B**) and naloxone (0.1 mg/ml; **C,D**) in SCI animals 8 weeks post treatment (12 weeks post injury) reduced the observed effect in the treated animals. Anti SHG reversed both tactile and cold allodynia attenuation; naloxone was effective in reversing cold allodynia (*p < 0.05, **p < 0.01, ***p < 0.001 vs. preinjection; ^#^*p* < 0.05, ^##^*p* < 0.01, ^###^*p* < 0.001 vs. GFP).

To further investigate the analgesic effect of SHG and EM1 in animals treated by compound construct, we have used some of the SCI animals from 6SHG/EM group for an intrathecal injection of anti-SHG (5 mg/ml; Figures 5A,B) and naloxone (0.1 mg/ml; Figures 5C,D) in order to block the effects of SHG and EM1, respectively. A catheter was inserted at 11 weeks post injury and the behavioral tests were performed at 12 weeks post injury. Drugs were injected at 2–5 days after pre-injection assessments for tactile and cold allodynia in this group. Injection of drugs led to partial reversal of the antinociceptive effects compared to GFP groups. Tactile and cold anti-allodynia were both partially reversed by anti-SHG in 6SHG/EM1 group (**p < 0.01, ***p < 0.001); no effect of this treatment was observed in the GFP group. Preinjection values in GFP and 6SHG/EM1 groups were significantly different (^###^*p* < 0.001); anti-SHG injection attenuated the effect of the treatment and the postinjection values were comparable to GFP group (Figures 5A,B). In cold allodynia test, the attenuation of the treatment effect was also observed although values did not reach the levels of control group. Naloxone injection did not affected tactile allodynia value, but partially reversed cold anti-allodynia in 6SHG/EM group (*p < 0.05). Pre and postinjection values between groups remained significantly different (^#^*p* < 0.05, ^###^*p* < 0.001, Figures 5C,D).

### Tissue Analysis

To confirm the expression of transgenes in the spinal cord, some spinal cord samples from CCI, SCI and control animals were prepared for neurochemical or immunohistochemical evaluation.

**Figure 6:**
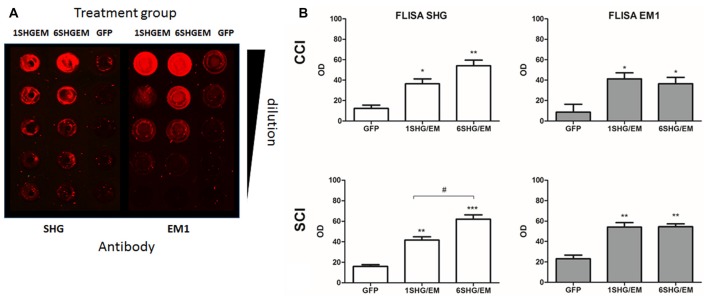
FLISA analysis of the spinal cord for the presence of recombinant SHG and EM1 peptides. **(A)** Both peptides were detected in the samples from 1SHG/EM1 and 6SHG/EM1 treated animals, with no signal from GFP treated animals. **(B)** OD signal of either peptide was enhanced in the samples from treated animals in both injury groups, with SHG signal significantly higher in 6SHG/EM1 samples compared to 1SHG/EM1 in SCI group. No differences between treatment samples for EM1 signal were observed as expected (ANOVA; *p < 0.05, **p < 0.01, ***p < 0.001 vs. GFP; ^#^*p* < 0.05 between treatments).

Spinal cord homogenates were analyzed for the presence of SHG and EM1 peptides by FLISA. Both peptides were detected in the samples from 1SHG/EM1 and 6SHG/EM1 treated animals, with no signal from GFP treated animals (Figure 6A). ANOVA analysis showed significant differences in the OD of SHG and EM1 peptides signal between treated and control groups in both injury models (Figure 6B; *p < 0.05, **p < 0.01). Enhanced SHG signal was observed in samples from 6SHG/EM1 treated animals, compared to 1SHG/EM1 in SCI group (^#^*p* < 0.05). The signal of EM1 was comparable between 1SHG/EM1 and 6SHG/EM1 samples (Figure 6B).

**Figure 7:**
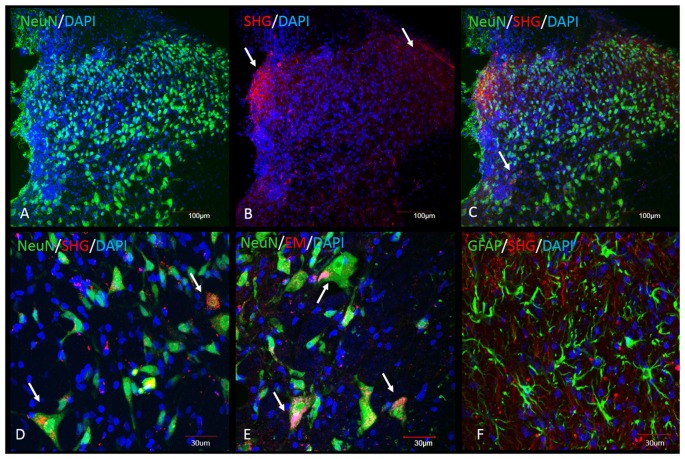
Immunohistochemical detection of recombinant SHG and EM1 peptides in the spinal cord of SCI animals. Both peptides were detected in the vicinity of the injection site with rostral and caudal spread within 1 mm. Top panel: Low magnification of spinal dorsal horn from animals after injection of the recombinant constructs. **(A)** NeuN and DAPI staining at the level of the injection site. SHG immunostaining was used to visualize the location of the construct. SHG positive fibers were detected in the lateral and medial side of the dorsal horn (arrows in **B**) and as a punctate cytoplasmic staining in NeuN cells (arrows in **C**). Bottom panel: Higher magnification of some of the neuronal and glial cells from the dorsal horn. Colocalization of NeuN with SHG **(D)** and EM1 **(E)** confirm transduction of neuronal cells (arrows). No significant colocalization with GFAP marker have been found **(F)**.

Immunohistochemical analysis was performed in the spinal cord tissue from SCI animals. Adjacent sections were processed for the staining with SHG and EM1 antibodies, as both antibodies were raised in the same host. SHG antibody was custom made by 21st Century Biochemicals, EM1 antibody was provided by Prof. Zadina. Both SHG and EM1 are not naturally present in the spinal tissue, therefore we assume the detected peptides are produced by injected gene constructs. Immunohistochemical analysis showed the presence of SHG and EM1 (Figure 7) The signal was detected in the vicinity of the injection site with rostral and caudal spread within 1 mm. Adjacent sections from thoracic (Th8–Th10) and cervical (C4–5) spinal cord were examined for the presence of recombinant peptides as well to confirm that spreading was limited to lumbar area only.

Transduced cells were observed throughout the dorsal horn, some signal was also detected around central canal. No significant staining was observed in the ventral horn, although we cannot rule out the possibility of transduction of cells in this area as well. The neurons primarily involved in the pain processing are located mostly in the superficial laminae, however deeper dorsal horn neurons in laminas LIII-V, the wide dynamic range neurons, are also involved in the pathological pain signaling. Transduction of both population of neurons is therefore beneficial in the attempt to induce analgesia using gene therapy approach.

Both peptides were located in the cytoplasm of the neurons or in the fibers in close vicinity of neurons.

## Discussion

This study evaluated the antinociceptive effect of compound gene constructs encoding the NMDA antagonist SHG and endogenous opioid EM1 in peripheral nerve injury and spinal cord injury pain models. Plasmids encoding single or multiple copies of SHG cDNA in combination with EM1 cDNA were engineered and subcloned into AAV2/8 viral particles. These constructs were injected intraspinally into animals with SCI or CCI induced pain. The results show that the engineered compound gene constructs are functional and are able to induce analgesia in both models. This is a first study using such approach and the positive outcome demonstrated by our results is promising for the further consideration of this approach as clinically applicable for the treatment of chronic pain.

Interference with the enhanced glutamate signaling via NMDA receptors after nerve injury or potentiation of mu-opioid receptor activation are among the most potent analgesic strategies. Many studies have shown attenuation of injury-induced hyperalgesia with NMDA antagonists (Suzuki et al., 2001; NasiriNezhad and Sagen, 2005; Kim et al., 2012) supporting the idea that increased excitatory signaling through NMDA receptors after nerve injury is one of the mechanisms underlying neuropathic pain. Opioids are historically the most potent analgesics. However, due to widespread location of the NMDA and opioid receptor within CNS, the long term use of either NMDA antagonist or opioids to manage chronic pain are associated with the development of tolerance, dependance, addiction and various side effects including constipation, motor disturbance and hallucinations (Hawksworth and Serpell, 1998). A strategy to overcome the burdens of chronic pain treatment is to develop a targeted delivery of analgesic substances to minimize the off-target effects, and to reduce the concentration of each of the active ingredients yet preserve the analgesic effects. The former is attempted by cell based or gene therapy approaches, the latter by using combinations of analgesic drugs with synergistic action at subthreshold concentrations.

Trujillo and Akil (1991) provided an initial evidence that NMDA antagonists may inhibit the development of morphine tolerance, thus allow prolonged administration of opioid-based analgesics. Since then, numerous studies confirmed this theory in experimental and clinical setting (Bilsky et al., 1996; Mao, 1999; Bisaga and Popik, 2000; Mendez and Trujillo, 2008; Lin et al., 2010; Vadivelu et al., 2016) and synergistic effects between opioids and NMDA antagonists towards pain relief has been demonstrated in several pain models (Nishiyama, 2000; Chow et al., 2004; Hama and Sagen, 2014). This approach has proven to be effective for combination of low-dose ketamine with opioids such as morphine, that showed more effective analgesic effect for pre or postoperative pain with minimal or no side effects. However, the analgesic potency of NMDA receptor antagonist and opioids combination varies in different pain states and also depends on the etiology of pain. For example, acute pain threshold in uninjured states is not changed by NMDA antagonists (Hoffmann et al., 2003; Redwine and Trujillo, 2003), but NMDA antagonists potentiates the analgesic effect of opioids in acute pain setting (Horvath et al., 2001; Hama and Sagen, 2014). On the other hand, the injury-induced pain is reduced by NMDA antagonists and the analgesic effect is further potentiated by combination with opioids (Hama and Sagen, 2014). Thus, the combination of these two targets may be particularly indicated for neural injury-induced pain. Our previous studies also shown that the route of delivery of the analgesic peptides into CNS can affect their overall action. Intrathecal injections of SHG and EMs reduced acute thermal pain and formalin-evoked hypersensitivity, but have marginal combination effects on CCI neuropathic pain symptoms. In contrast, co-injection of a mixture of lentivectors encoding SHG and EM1/EM2 significantly attenuated neuropathic pain related behavior after SCI (NasiriNezhad et al., 2015). These results may indicate that intrathecal administration of these peptides is not the ideal route for co-delivery due to possible different pharmacokinetics, diffusion and peak of activity. Local production of analgesic peptides by transgenes inserted into CNS may overcome these issues.

This is supported by findings of the current study using compound gene constructs encoding these analgesic peptides. Intraspinal injection of single and multicopy SHG and single EM1 gene reduced tactile and cold allodynia in CCI and SCI models.

Injections of the constructs were done at the lumbar enlargement in both models, despite the thoracic location of the SCI. For CCI model, lumbar spinal segments are the primary centers of the neuropatic changes. In SCI model, the rationale was to target a primary clinical concern for SCI patients, which is below-level neuropathic pain, frequently reported as the most severe and bothersome by these patients (Siddall et al., 1995, 1997, 1999; Widerström-Noga et al., 2001; Cruz-Almeida et al., 2009). In addition, several studies have shown that there are significant changes in the spinal dorsal horn at levels far removed from the actual injury site, suggesting that aberrant signaling in those regions may underlie or contribute to below-level SCI pain (Detloff et al., 2008; Gwak and Hulsebosch, 2009; Gwak et al., 2012; Redondo-Castro et al., 2013). Further, this target site allows us to utilize standard hind-paw tests for allodynia and hyperalgesia, mediated at the L3–L5 level (Asato et al., 2000).

The interesting finding was a different potency of the treatment depending on the pain modality and type of injury. In the CCI model, the effect of either construct was stronger in reducing cold allodynia, with only modest reduction in tactile allodynia. In the SCI model, injection of the constructs led to attenuation of pain like behavior in both pain modalities, tactile and cold allodynia, with the strongest effects shown by the 6SHG construct.

The differences in the efficacy of our treatment between CCI and SCI model as indicated by our results may be caused by several factors. One of the hypothesized mechanisms is related to disruption in descending inhibition in SCI model. Nociceptive signal from periphery conveyed via spinothalamic tract (STT) activates descending inhibitory control (Bruce et al., 2002). Damage of STT after SCI may affect descending inhibition and cause prolonged central pain due to hyperactivity of the spinal neurons. While STT tract is unaffected by peripheral nerve injury, it is possible that overall activity of spinal NMDA receptors is lower than in the SCI model and thus descending inhibitory input may be partially responsible for less pronounced analgesic effect of our treatment in the model of peripheral nerve injury compared to SCI.

While anti-allodynic effects of the individual peptide constructs were promising, neither of these alone completely reversed tactile or cold allodynia. To enhance the therapeutic potential of our approach, and to further explore the potency of different combinations of our analgesic constructs, we have designed compound gene constructs encoding SHG and EM1 peptides. Our results demonstrated that the 6SHG/EM1 combined construct is the most potent in reducing pain like behavior in both CCI and SCI models. The observed analgesic effect was stable over several weeks, perhaps permanently, especially in the SCI model of chronic pain. The stable analgesic effect over a prolonged period of time observed in SCI animals further support the finding of previous studies, that NMDA receptor antagonists prevent development of opioid tolerance (Nishiyama, 2000; Chow et al., 2004). The active involvement of the recombinant peptides in the nociceptive processing in treated animals is also supported by the pharmacological reversal of the behavior after intrathecal injection of anti-SHG or naloxone demonstrated in this study.

Tissue analysis demonstrated the presence of both SHG and EM1 recombinant peptides in the spinal cord. As SHG is not naturally presented in the spinal cord, the detected peptide is a result of its active production by inserted gene construct. Similarly, EM1 isoform is mostly presented in the brain tissue (Zadina et al., 1999), with EM2 reported as the main isoform in the spinal tissue. The specificity of EM1 antibody used for immunohistochemical and biochemical detections in this study (courtesy of Prof. Zadina) has been previously demonstrated (Martin-Schild et al., 1999). Immunohistochemistry confirmed the presence of recombinant peptides in close vicinity of the injection site. These findings further support the benefits of a gene therapy as a targeted approach as recombinant peptides were not observed in the remote areas, thus therapeutic effects may be directed more precisely to desired sites.

The results from this study provide additional evidence that gene therapy is a suitable tool to introduce analgesic peptides into the CNS.

Previous studies showed that the effect of intrathecal SHG and EM varied depending on outcome measure, likely because of the labile nature of the small neuropeptides in the CSF (Hama and Sagen, 2014). As this study and our previous study show, gene therapy offers longer effects and better efficacy than introduction of analgesic peptides intrathecally (NasiriNezhad et al., 2015). Additionally, direct introduction of analgesic compounds avoids the unwanted side effects of systemic pain therapy.

The AAV vector is ideal for neuronal delivery of cDNA constructs as it efficiently transduces neuronal targets, and offers long-term expression of transgenes encoded within the vector (Mitchell et al., 2010). Moreover, studies have shown little to no immune or inflammatory response to AAV administration into the CNS (Chamberlin et al., 1998; Mastakov et al., 2002).

However, the widespread exposure of human population to AAVs and the preexisting immunity to various serotypes is one of the challenges to overcome in clinical trials. The extensive research on this issue showed that modification of the capsid proteins or immunogenic epitopes may reduce immune clearance (Mingozzi et al., 2013). Several clinical trials for neurodegenerative diseases are currently ongoing using AAV as a carrier (Piguet et al., 2017). Potential safety concerns of using AAV vectors regarding mutagenesis have been addressed by several studies indicating that AAV vectors are generally safe. However, insertional mutagenesis has been reported in one study following mice neonatal injection of AAV vector containing CMV enhancer, chicken B actin promoter and human beta-glucoronidase gene (Donsante et al., 2007). Safety evaluations will thus be needed for translational studies, including long-term observation of the transgene effect and identification of possible tumorigenic components of the vector.

Gene therapy thus offers sustained, targeted activation of analgesic pathways in the central nervous system. Furthermore, by forming novel compound combinations and constructing cDNAs encoding these combinations, gene therapy allows one to simultaneously activate different signaling pathways using multiple antinociceptive peptides.

## Conclusion

In conclusion, this study provides evidence for the use of intraspinal gene therapy with a combination of NMDA antagonists and endogenous opioids for the treatment of neuropathic pain. The injection of AAV vectors encoding combined SHG-EM constructs leads to long term attenuation of pain without overt adverse side effects. Gene therapy with novel compounds can overcome the limitations of traditional pharmacotherapy for patients suffering from chronic disabling pain.

## Author Contributions

SJ designed and conducted all experiments and data analysis, drafted the manuscript. CEG conducted gene engineering, data analysis and drafted the manuscript. SG helped with gene design, manuscript draft. JS contributed with advices on data analysis, final revision and discussion of manuscript.

## Conflict of Interest Statement

The authors declare that the research was conducted in the absence of any commercial or financial relationships that could be construed as a potential conflict of interest.

## References

[B1] Albert K. Voutilainen M. H. Domanskyi A. Airavaara M. (2017). AAV vector-mediated gene delivery to substantia nigra dopamine neurons: implications for gene therapy and disease models. Genes 8:E63. 10.3390/genes8020063 28208742 PMC5333052

[B2] Asato F. Butler M. Blomberg H. Gordh T. (2000). Variation in rat sciatic nerve anatomy: implications for a rat model of neuropathic pain. J. Peripher. Nerv. Syst. 5, 19–21. 10.1046/j.1529-8027.2000.00155.x 10780679

[B3] Bennett G. J. Xie Y. K. (1988). A peripheral mononeuropathy in rat that produces disorders of pain sensation like those seen in man. Pain 33, 87–107. 10.1016/0304-3959(88)90209-6 2837713

[B4] Beutler A. S. Reinhardt M. (2009). AAV for pain: steps towards clinical translation. Gene Ther. 16, 461–469. 10.1038/gt.2009.23 19262609

[B5] Beutler A. S. Banck M. S. Walsh C. E. Milligan E. D. (2005). Intrathecal gene transfer by adeno-associated virus for pain. Curr. Opin. Mol. Ther. 7, 431–439. 16248278

[B6] Bilsky E. J. Inturrisi C. E. Sadee W. Hruby V. J. Porreca F. (1996). Competitive and non-competitive NMDA antagonists block the development of antinociceptive tolerance to morphine, but not to selective mu or delta opioid agonists in mice. Pain 68, 229–237. 10.1016/s0304-3959(96)03185-5 9121809

[B7] Bisaga A. Popik P. (2000). In search of a new pharmacological treatment for drug and alcohol addiction: *N*-methyl-D-aspartate (NMDA) antagonists. Drug Alcohol Depend. 59, 1–15. 10.1016/s0376-8716(99)00107-6 10706971

[B8] Bruce J. C. Oatway M. A. Weaver L. C. (2002). Chronic pain after clip-compression injury of the rat spinal cord. Exp. Neurol. 178, 33–48. 10.1006/exnr.2002.8026 12460606

[B9] Chamberlin N. L. Du B. de Lacalle S. Saper C. B. (1998). Recombinant adeno-associated virus vector: use for transgene expression and anterograde tract tracing in the CNS. Brain Res. 793, 169–175. 10.1016/s0006-8993(98)00169-3 9630611 PMC4961038

[B10] Chaplan S. R. Bach F. W. Pogrel J. W. Chung J. M. Yaksh T. L. (1994). Quantitative assessment of tactile allodynia in the rat paw. J. Neurosci. Methods 53, 55–63. 10.1016/0165-0270(94)90144-9 7990513

[B11] Choi Y. Yoon Y. W. Na H. S. Kim S. H. Chung J. M. (1994). Behavioral signs of ongoing pain and cold allodynia in a rat model of neuropathic pain. Pain 59, 369–376. 10.1016/0304-3959(94)90023-x 7708411

[B12] Chow L. H. Huang E. Y. Ho S. T. Lee T. Y. Tao P. L. (2004). Dextromethorphan potentiates morphine antinociception at the spinal level in rats. Can. J. Anaesth. 51, 905–910. 10.1007/bf03018888 15525615

[B13] Cruz-Almeida Y. Felix E. R. Martinez-Arizala A. Widerström-Noga E. G. (2009). Pain symptom profiles in persons with spinal cord injury. Pain Med. 10, 1246–1259. 10.1111/j.1526-4637.2009.00713.x 19818035

[B14] Cruz-Almeida Y. Martinez-Arizala A. Widerström-Noga E. G. (2005). Chronicity of pain associated with spinal cord injury: a longitudinal analysis. J. Rehabil. Res. Dev. 42, 585–594. 10.1682/jrrd.2005.02.0045 16586184

[B15] Detloff M. R. Fisher L. C. McGaughy V. Longbrake E. E. Popovich P. G. Basso D. M. (2008). Remote activation of microglia and pro-inflammatory cytokines predict the onset and severity of below-level neuropathic pain after spinal cord injury in rats. Exp. Neurol. 212, 337–347. 10.1016/j.expneurol.2008.04.009 18511041 PMC2600773

[B16] Donsante A. Miller D. G. Li Y. Vogler C. Brunt E. M. Russell D. W.. (2007). AAV vector integration sites in mouse hepatocellular carcinoma. Science 317:477. 10.1126/science.1142658 17656716

[B17] Dugan E. A. Sagen J. (2015a). “An over ground testing apparatus for evaluation of spinal cord injury associated neuropathic pain,” in Society for Neuroscience, 63.16/M3.

[B18] Dugan E. A. Sagen J. (2015b). An intensive locomotor training paradigm improves neuropathic pain following spinal cord compression injury in rats. J. Neurotrauma 32, 622–632. 10.1089/neu.2014.3692 25539034

[B19] Duplan H. Li R. Y. Vue C. Zhou H. Emorine L. Herman J. P.. (2004). Grafts of immortalized chromaffin cells bio-engineered to improve met-enkephalin release also reduce formalin-evoked c-fos expression in rat spinal cord. Neurosci. Lett. 370, 1–6. 10.1016/j.neulet.2004.07.017 15489007

[B20] Eaton M. J. Blits B. Ruitenberg M. J. Verhaagen J. Oudega M. (2002). Amelioration of chronic neuropathic pain after partial nerve injury by adeno-associated viral (AAV) vector-mediated over-expression of BDNF in the rat spinal cord. Gene Ther. 9, 1387–1395. 10.1038/sj.gt.3301814 12365004

[B21] Finnerup N. B. Baastrup C. (2012). Spinal cord injury pain: mechanisms and management. Curr. Pain Headache Rep. 16, 207–216. 10.1007/s11916-012-0259-x 22392531

[B22] Gajavelli S. Castellanos D. A. Furmanski O. Schiller P. C. Sagen J. (2008). Sustained analgesic peptide secretion and cell labeling using a novel genetic modification. Cell Transplant. 17, 445–455. 18522246 PMC2743252

[B23] Galluzzi K. E. (2005). Management of neuropathic pain. J. Am. Osteopath. Assoc. 105, S12–S19. 16273720

[B24] Guedon J. M. Wu S. Zheng X. Churchill C. C. Glorioso J. C. Liu C. H.. (2015). Current gene therapy using viral vectors for chronic pain. Mol. Pain 11:27. 10.1186/s12990-015-0018-1 25962909 PMC4446851

[B25] Gwak Y. S. Hulsebosch C. E. (2009). Remote astrocytic and microglial activation modulates neuronal hyperexcitability and below-level neuropathic pain after spinal injury in rat. Neuroscience 161, 895–903. 10.1016/j.neuroscience.2009.03.055 19332108 PMC3005301

[B26] Gwak Y. S. Kang J. Unabia G. C. Hulsebosch C. E. (2012). Spatial and temporal activation of spinal glial cells: role of gliopathy in central neuropathic pain following spinal cord injury in rats. Exp. Neurol. 234, 362–372. 10.1016/j.expneurol.2011.10.010 22036747 PMC3303938

[B27] Hama A. Sagen J. (2002). Selective antihyperalgesic effect of [Ser1] histogranin on complete Freund’s adjuvant-induced hyperalgesia in rats. Pain 95, 15–21. 10.1016/s0304-3959(01)00368-2 11790463

[B28] Hama A. Sagen J. (2007). Behavioral characterization and effect of clinical drugs in a rat model of pain following spinal cord compression. Brain Res. 1185, 117–128. 10.1016/j.brainres.2007.09.013 17935699

[B29] Hama A. Sagen J. (2009). Sustained antinociceptive effect of cannabinoid receptor agonist WIN 55,212–2 over time in rat model of neuropathic spinal cord injury pain. J. Rehabil. Res. Dev. 46, 135–143. 10.1682/JRRD.2008.04.0049 19533526 PMC2743245

[B30] Hama A. Sagen J. (2014). Selective antinociceptive effects of a combination of the *N*-methyl-D-aspartate receptor peptide antagonist [Ser^1^]histogranin and morphine in rat models of pain. Pharmacol. Res. Perspect. 2:e00032. 10.1002/prp2.32 25505581 PMC4184704

[B31] Hama A. T. Siegan J. B. Herzberg U. Sagen J. (1999). NMDA-induced spinal hypersensitivity is reduced by naturally derived peptide analog [Ser 1]histogranin. Pharmacol. Biochem. Behav. 62, 67–74. 10.1016/s0091-3057(98)00132-4 9972847

[B32] Hawksworth C. Serpell M. (1998). Intrathecal anesthesia with ketamine. Reg. Anesth. Pain Med. 23, 283–288. 10.1097/00115550-199823030-00010 9613541

[B33] Hentall I. D. Hargraves W. A. Sagen J. (2007). Inhibition by the chromaffin cell-derived peptide serine-histogranin in the rat’s dorsal horn. Neurosci. Lett. 419, 88–92. 10.1016/j.neulet.2007.03.056 17442490 PMC1945824

[B34] Hoffmann V. L. Baker A. K. Vercauteren M. P. Adriaensen H. F. Meert T. F. (2003). Epidural ketamine potentiates epidural morphine but not fentanyl in acute nociception in rats. Eur. J. Pain 7, 121–130. 10.1016/s1090-3801(02)00074-5 12600793

[B35] Horvath G. Joo G. Dobos I. Klimscha W. Toth G. Benedek G. (2001). The synergistic antinociceptive interactions of endomorphin-1 with dexmedetomidine and/or S(+)-ketamine in rats. Anesth. Analg. 93, 1018–1024. 10.1097/00000539-200110000-00044 11574376

[B36] Jergova S. Gajavelli S. Pathak N. Sagen J. (2016a). Recombinant neural progenitor transplants in the spinal dorsal horn alleviate chronic central neuropathic pain. Pain 157, 977–989. 10.1097/j.pain.0000000000000471 26761378 PMC4938010

[B37] Jergova S. Gajavelli S. Varghese M. S. Shekane P. Sagen J. (2016b). Analgesic Effect of recombinant GABAergic cells in a model of peripheral neuropathic pain. Cell Transplant. 25, 629–643. 10.3727/096368916x690782 26817412

[B38] Jergova S. Hentall I. D. Gajavelli S. Varghese M. S. Sagen J. (2012). Intraspinal transplantation of GABAergic neural progenitors attenuates neuropathic pain in rats: a pharmacologic and neurophysiological evaluation. Exp. Neurol. 234, 39–49. 10.1016/j.expneurol.2011.12.005 22193109 PMC3294082

[B39] Kim Y. Cho H. Y. Ahn Y. J. Kim J. Yoon Y. W. (2012). Effect of NMDA NR2B antagonist on neuropathic pain in two spinal cord injury models. Pain 153, 1022–1029. 10.1016/j.pain.2012.02.003 22424878

[B40] Kotterman A. M. Schaffer D. V. (2015). Engineered AAV vectors for improved central nervous system gene delivery. Neurogenesis 2:e1122700. 10.1080/23262133.2015.1122700 27606332 PMC4973602

[B41] Lemaire S. Rogers C. Dumont M. Shukla V. K. Lapierre C. Prasad J.. (1995). Histogranin, a modified histone H4 fragment endowed with N-methyl-D-aspartate antagonist and immunostimulatory activities. Life Sci. 56, 1233–1241. 10.1016/0024-3205(95)00068-2 8614240

[B42] Lemaire S. Shukla V. K. Rogers C. Ibrahim I. H. Lapierre C. Parent P.. (1993). Isolation and characterization of histogranin, a natural peptide with NMDA receptor antagonist activity. Eur. J. Pharmacol. 245, 247–256. 10.1016/0922-4106(93)90104-h 8101490

[B43] Lin S.-L. Tsai R.-Y. Shen C.-H. Lin F.-H. Wang J.-J. Hsin S.-T.. (2010). Co-administration of ultra-low dose naloxone attenuates morphine tolerance in rats via attenuation of NMDA receptor neurotransmission and suppression of neuroinflammation in the spinal cords. Pharmacol. Biochem. Behav. 96, 236–245. 10.1016/j.pbb.2010.05.012 20478329

[B44] Mao J. (1999). NMDA and opioid receptors: their interactions in antinociception, tolerance and neuroplasticity. Brain Res. Rev. 30, 289–304. 10.1016/s0165-0173(99)00020-x 10567729

[B45] Martin-Schild S. Gerall A. A. Kastin A. J. Zadina J. E. (1999). Differential distribution of endomorphin 1- and endomorphin 2-like immunoreactivities in the CNS of the rodent. J. Comp. Neurol. 405, 450–471. 10.1002/(sici)1096-9861(19990322)405:4<450::aid-cne2>3.0.co;2-# 10098939

[B46] Mastakov M. Y. Baer K. Symes C. W. Leichtlein C. B. Kotin R. M. During M. J. (2002). Immunological aspects of recombinant adeno-associated virus delivery to the mammalian brain. J. Virol. 76, 8446–8454. 10.1128/jvi.76.16.8446-8454.2002 12134047 PMC155154

[B47] Mendez I. A. Trujillo K. A. (2008). NMDA receptor antagonists inhibit opiate antinociceptive tolerance and locomotor sensitization in rats. Psychopharmacology 196, 497–509. 10.1007/s00213-007-0984-8 17994223

[B48] Mingozzi F. Anguela X. M. Pavani G. Chen Y. Davidson R. J. Hui D. J.. (2013). Overcoming preexisting humoral immunity to AAV using capsid decoys. Sci. Transl. Med. 5:194ra192. 10.1126/scitranslmed.3005795 23863832 PMC4095828

[B49] Mitchell A. M. Nicolson S. C. Warischalk J. K. Samulski R. J. (2010). AAV’s anatomy: roadmap for optimizing vectors for translational success. Curr. Gene Ther. 10, 319–340. 10.2174/156652310793180706 20712583 PMC3920455

[B51] NasiriNezhad F. Gajavelli S. Priddy B. Jergova S. Zadina J. Sagen J. (2015). Viral vectors encoding endomorphins and serine histogranin attenuate neuropathic pain symptoms after spinal cord injury in rats. Mol. Pain 11:2. 10.1186/1744-8069-11-2 25563474 PMC4349602

[B50] NasiriNezhad F. Sagen J. (2005). NMDA antagonist peptide supplementation enhances pain alleviation by adrenal medullary transplants. Cell Transplant. 14, 203–211. 10.3727/0000000057800013983115 15929555

[B52] Naso M. F. Tomkowicz B. Perry W. L. III Strohl W. R. (2017). Adeno-associated virus (AAV) as a vector for gene therapy. BioDrugs [Epub ahead of print]. 10.1007/s40259-017-0234-5 28669112 PMC5548848

[B53] Nishiyama T. (2000). Interaction between intrathecal morphine and glutamate receptor antagonists in formalin test. Eur. J. Pharmacol. 395, 203–210. 10.1016/s0014-2999(00)00268-5 10812050

[B54] Ossipov M. H. Lai J. King T. Vanderah T. W. Malan T. P. Jr. Hruby V. J.. (2004). Antinociceptive and nociceptive actions of opioids. J. Neurobiol. 61, 126–148. 10.1002/neu.20091 15362157

[B55] Piguet F. Alves S. Cartier N. (2017). Clinical gene therapy for neurodegenerative diseases: past, present, and future. Hum. Gene Ther. 28, 988–1003. 10.1089/hum.2017.160 29035118

[B56] Pleticha J. Heilmann L. F. Evans C. H. Asokan A. Samulski R. J. Beutler A. S. (2014). Preclinical toxicity evaluation of AAV for pain: evidence from human AAV studies and from the pharmacology of analgesic drugs. Mol. Pain 10:54. 10.1186/1744-8069-10-54 25183392 PMC4237902

[B57] Prasad J. A. Shukla V. K. Lemaire S. (1995). Synthesis and biological activity of histogranin and related peptides. Can. J. Physiol. Pharmacol. 73, 209–214. 10.1139/y95-030 7621358

[B58] Przewłocka B. Mika J. Labuz D. Toth G. Przewłocki R. (1999). Spinal analgesic action of endomorphins in acute, inflammatory and neuropathic pain in rats. Eur. J. Pharmacol. 367, 189–196. 10.1016/s0014-2999(98)00956-x 10078992

[B60] Przewłocki R. Labuz D. Mika J. Przewłocka B. Tomboly C. Toth G. (1999). Pain inhibition by endomorphins. Ann. N Y Acad. Sci. 897, 154–164. 10.1111/j.1749-6632.1999.tb07887.x 10676444

[B59] Przewłocki R. Przewłocka B. (2001). Opioids in chronic pain. Eur. J. Pharmacol. 429, 79–91. 11698029 10.1016/s0014-2999(01)01308-5

[B61] Redondo-Castro E. García-Alías G. Navarro X. (2013). Plastic changes in lumbar segments after thoracic spinal cord injuries in adult rats: an integrative view of spinal nociceptive dysfunctions. Restor. Neurol. Neurosci. 31, 411–430. 10.3233/RNN-120291 23612035

[B62] Redwine K. E. Trujillo K. A. (2003). Effects of NMDA receptor antagonists on acute μ-opioid analgesia in the rat. Pharmacol. Biochem. Behav. 76, 361–372. 10.1016/j.pbb.2003.08.009 14592689

[B63] Sallerin-Caute B. Lazorthes Y. Deguine O. Frances B. Verdie J. C. Charlet J. P.. (1998). Does intrathecal morphine in the treatment of cancer pain induce the development of tolerance? Neurosurgery 42, 44–49; discussion 49–50. 10.1097/00006123-199801000-00009 9442502

[B64] Siddall P. J. Taylor D. Cousins M. J. (1995). Pain associated with spinal cord injury. Curr. Opin. Neurol. 8, 447–450. 8845929 10.1097/00019052-199512000-00009

[B65] Siddall P. J. Taylor D. A. Cousins M. J. (1997). Classification of pain following spinal cord injury. Spinal Cord 35, 69–75. 10.1038/sj.sc.3100365 9044512

[B66] Siddall P. J. Taylor D. A. McClelland J. M. Rutkowski S. B. Cousins M. J. (1999). Pain report and the relationship of pain to physical factors in the first 6 months following spinal cord injury. Pain 81, 187–197. 10.1016/s0304-3959(99)00023-8 10353507

[B67] Siegan J. B. Hama A. T. Sagen J. (1997). Suppression of neuropathic pain by a naturally-derived peptide with NMDA antagonist activity. Brain Res. 755, 331–334. 10.1016/s0006-8993(97)00183-2 9175901

[B68] Sotgiu M. L. Biella G. (2000). Differential effects of MK-801, a *N*-methyl-D-aspartate non-competitive antagonist, on the dorsal horn neuron hyperactivity and hyperexcitability in neuropathic rats. Neurosci. Lett. 283, 153–156. 10.1016/s0304-3940(00)00941-1 10739898

[B69] Suzuki R. Matthews E. A. Dickenson A. H. (2001). Comparison of the effects of MK-801, ketamine and memantine on responses of spinal dorsal horn neurones in a rat model of mononeuropathy. Pain 91, 101–109. 10.1016/s0304-3959(00)00423-1 11240082

[B70] Taylor K. S. Anastakis D. J. Davis K. D. (2010). Chronic pain and sensorimotor deficits following peripheral nerve injury. Pain 151, 582–591. 10.1016/j.pain.2010.06.032 20655145

[B71] Trujillo K. A. Akil H. (1991). Inhibition of morphine tolerance and dependence by the NMDA receptor antagonist MK-801. Science 251, 85–87. 10.1126/science.1824728 1824728

[B72] Trujillo K. A. Akil H. (1994). Inhibition of opiate tolerance by non-competitive N-methyl-D-aspartate receptor antagonists. Brain Res. 633, 178–188. 10.1016/0006-8993(94)91538-5 8137155

[B73] Vadivelu N. Schermer E. Kodumudi V. Belani K. Urman R. D. Kaye A. D. (2016). Role of ketamine for analgesia in adults and children. J. Anaesthesiol. Clin. Pharmacol. 32, 298–306. 10.4103/0970-9185.168149 27625475 PMC5009833

[B74] Wells D. J. (2017). Systemic AAV gene therapy close to clinical trials for several neuromuscular diseases. Mol. Ther. 25, 834–835. 10.1016/j.ymthe.2017.03.006 28341564 PMC5383641

[B75] Widerström-Noga E. G. Felipe-Cuervo E. Yezierski R. P. (2001). Chronic pain after spinal injury: interference with sleep and daily activities. Arch. Phys. Med. Rehabil. 82, 1571–1577. 10.1053/apmr.2001.26068 11689978

[B77] Yaksh T. L. Fisher C. J. Hockman T. M. Wiese A. J. (2017). Current and future issues in the development of spinal agents for the management of pain. Curr. Neuropharmacol. 15, 232–259. 10.2174/1570159x14666160307145542 26861470 PMC5412694

[B76] Yaksh T. L. Rudy T. A. (1976). Chronic catheterization of the spinal subarachnoid space. Physiol. Behav. 17, 1031–1036. 10.1016/0031-9384(76)90029-9 14677603

[B78] Zadina J. E. Martin-Schild S. Gerall A. A. Kastin A. J. Hackler L. Ge L. J.. (1999). Endomorphins: novel endogenous mu-opiate receptor agonists in regions of high mu-opiate receptor density. Ann. N Y Acad. Sci. 897, 136–144. 10.1111/j.1749-6632.1999.tb07885.x 10676442

